# Conservative interventions for incontinence in people with dementia or cognitive impairment, living at home: a systematic review

**DOI:** 10.1186/1471-2318-12-77

**Published:** 2012-12-28

**Authors:** Vari M Drennan, Nan Greenwood, Laura Cole, Mandy Fader, Robert Grant, Greta Rait, Steve Iliffe

**Affiliations:** 1 Faculty of Health & Social Care Sciences, St. George’s University of London & Kingston University, Cranmer Terrace, London SW170RE, United Kingdom (UK; 2 Faculty of Health Sciences, Southampton University, High field, Southampton, SO17 1BJ, UK; 3 PRIMENT Clinical Trials Unit, Research Department of Primary Care and Population Health, University College London Medical School (Royal Free Campus), Rowland Hill Street, London, NW3 2PF, UK; 4 Research Department of Primary Care and Population Health, University College London Medical School (Royal Free Campus), Rowland Hill Street, London, NW3 2PF, UK

**Keywords:** Dementia, Cognitive impairment, Incontinence, Community dwelling, Review, Carer/caregiver

## Abstract

**Background:**

Dementia is a distressing and disabling illness with worldwide estimates of increased numbers of people with the condition. Two thirds of people with dementia live at home and policies in many countries seek to support more people for longer in this setting. Incontinence both contributes to carer burden and is also a significant factor in the decision to move into care homes. A review was conducted for evidence of effectiveness for conservative interventions, which are non-pharmacological and non-surgical interventions, for the prevention or management of incontinence in community dwelling people with dementia.

**Method:**

Fourteen electronic databases were searched, including MEDLINE, EMBASE and CINAHL (from inception to 2012). Assessments of risk of bias were made. Meta-analysis was inappropriate due to the heterogeneity of the interventions and outcome measurements. A narrative analysis was undertaken.

**Results:**

From 427 identified abstracts, 56 studies were examined but only three met the inclusion criteria, all more than a decade old. All three focused on urinary incontinence. Two studies were exploratory or pilot studies. All had a control arm. The interventions were of advice for the carer to implement. Two included toileting education of prompted voiding or an individualised toileting schedule. There was insufficient evidence to support or rule out effectiveness of any of these interventions. Some interventions were unacceptable for some carers. None specifically reported the perspective of the person with dementia.

**Conclusions:**

There was insufficient evidence from any studies to recommend any strategies. There remains an urgent need for both research and also clinical guidance for health professionals tailored to community settings where the majority of people with dementia live.

## Background

Dementia is one of the most disabling and onerous diseases. Estimates suggest that up to six million people worldwide receive a diagnosis each year [1], with predications of future increased numbers and impact for all health care systems [2]. There are many who will not receive a diagnosis of dementia until late in the course of the disease for a variety of reasons [3] but have recognisable cognitive deficits. Mild cognitive impairment is used to describe those with measurable cognitive deficits, without a dementia diagnosis but thought to be at high risk of progressing to a dementia disorder [4]. Two thirds of people with dementia live at home [5]. Clinicians and health service planners are seeking ways to enable more patients to remain longer at home, as advocated in national dementia strategies [6-8].

The dementia syndromes result in deterioration in: cognition, abilities to undertake activities of daily living including personal toileting, and physical functioning [9]. In addition behavioural and psychological symptoms can result in inappropriate voiding [10]. Cognitive impairment resulting in specific cortical abnormalities is also known to be a factor in geriatric urge incontinence [11]. Incontinence contributes to carer burden [12] and is significant in decisions to move into care homes [13]. Carers, in this paper referring to family and other informal caregivers, describe a range of problems and their impact [14]. Guidelines for the support and management of people with dementia often provide little detail on incontinence [see for example [15]. Internationally agreed algorithms provide guidance on the assessment and treatment of urinary incontinence (UI) and faecal incontinence (FI) [16]. However guidelines often exclude patients with dementia see for example [17,18] or subsume them within the frail elderly [19]. Incontinence in older adults has been described as a geriatric syndrome [20] with multiple underlying factors, and as such interventions are likely to require “human capital, rather than simply a new drug or technology” [21] p787. Conservative management of incontinence has been defined as “any therapy that does not involve pharmacological or surgical intervention” [22] p1020, for example behavioural therapies. Behavioural therapies such as prompted voiding have been described as the mainstay of UI in groups such as the frail elderly [19] although there is little differentiation between the setting of care home and an individual residence. The setting is, however, important in considering the feasibility and effectiveness of conservative interventions for the prevention of incontinence and management of incontinence. At the very least the domestic environment may not be designed or adaptable for people with disabilities and there is likely to be a different level of availability of (able bodied and fit) people to assist. Also individual preferences, including of those living in the same household, will be paramount. An individual’s or their relative’s home creates a very different setting to a care home. People with dementia or cognitive impairment and incontinence problems, living at home, are likely to require tailored, evidence based, interventions and advice from their generalist primary care and specialist health professionals. We, therefore, conducted a systematic review to assess the effectiveness of conservative interventions for the prevention or management of incontinence in community dwelling people with dementia or cognitive impairment.

The review sought studies providing empirical data from randomised, quasi randomised or observational studies of interventions. The participants were defined as people with dementia or cognitive impairment, living at home, with incontinence problems. The interventions of interest were conservative measures i.e. non surgical and non pharmacological. The main outcomes of interest were the effect on: episodes of incontinence, burden for carers, quality of life for people with dementia and carers, and on costs. In addition, any data on the acceptability (or otherwise) of the intervention was of interest.

## Methods

A systematic review was undertaken following the Cochrane review methods [23]. A search for papers in the following electronic databases was carried out, MEDLINE, EMBASE, CINAHL, PsycINFO, BNI, CAREDATA and the Cochrane Library (including DARE, NTIS, SIGLE), Social Science Citation Index, Age Info, National Research Register, Papers First and the specialised register of the Cochrane Effective Practice and Organisation of Care Group (EPOC), and Dissertation Abstracts. The data bases were searched from the start date e.g. MEDLINE 1950 to 2012 week 13 (4^th^ April): Medical search headings and key words (Table 1) were used in combination and an example electronic search strategy is presented in Additional file 1. In addition, lateral searching techniques were used for key authors and cited references.

**Table 1:** Search terms

**Area**	**Search terms (medical subject headings and key words)**
*Population characteristics*	Delirium, Dementia, Amnestic, Cognitive Disorders/ or exp Dementia/. Dementia. Aged. Elderly
*Setting*	Community dwelling. Community. Community living. Homebound patients.
*Research field of enquiry*	Urinary Incontinence. Fecal Incontinence.
	Self care, Activities of daily living or adl. Toilet. Toilet facilities. Toilet training. Behavior therapy. Incontinence pads, diapers.
	Caregivers, Caregiver burden, Spouses, Family.
	Ambulatory care, Ambulatory care nursing, Home health care, Home health agencies, Home nursing, Community health services, Home care services, Social support, Occupational therapy

Abstracts were screened by two researchers [NG,VMD] using the following inclusion criteria: intervention studies addressing problems of incontinence (UI and or FI) experienced by people with dementia or cognitive impairments living at home and reported in English. Studies were excluded if they did not report empirical data, were set in hospital, nursing, care or group residential homes, or excluded people with cognitive impairment or dementia or where they were included but findings were not reported separately. For ambiguous abstracts, the full text papers were retrieved and read. Data were extracted against pre-defined categories by one researcher and confirmed by a second researcher [NG, LC,VMD]. These categories were: date of publication, country of study, study design, characteristics of participants, methods of determining dementia or cognitive impairment and incontinence, attrition, the intervention, the follow up period, outcomes including measurements of incontinence, burden for carers, quality of life for people with dementia and carers, and costs of the intervention. Each included study was assessed against the five domains of the Cochrane risk of bias tool [24].

## Results

**Figure 1:**
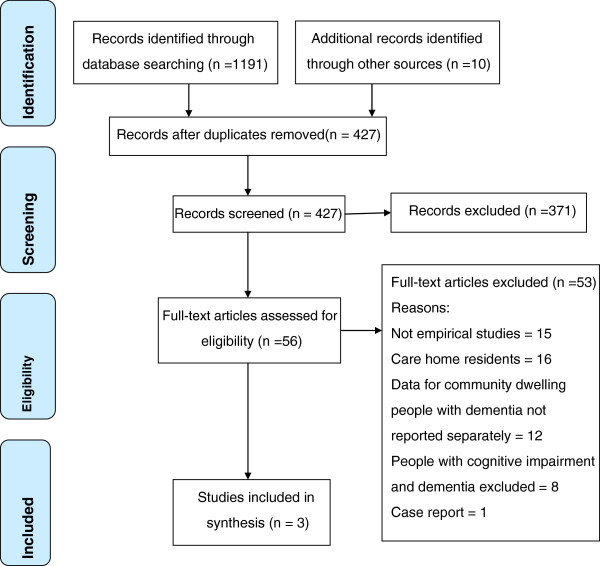
**PRISMA [**[25]**] Flow diagram of search results.**

A total of 427 abstracts were identified and 56 studies retained for full text reading (Figure 1). We report the review using the framework provided by the PRISMA (Preferred Reporting Items for Systematic Reviews and Meta-Analyses) Statement Group [25]. After scrutiny, 53 were excluded and details are included in Figure 1. Three studies were included in the review. The heterogeneity of the interventions and outcomes made a meta-analysis inappropriate. A narrative summary of findings is therefore presented. Characteristics of the three studies are presented in Table 2.

**Table 2:** Characteristics of the included studies

**Authors, date & location**	**Research question**	**Study design**	**Participants: recruitment method, eligibility, characteristics and attrition**	**Intervention and control**	**Outcome measure**
Gitlin and Corcoran [26] 1993 USA Community	To test the effectiveness of a home based intervention to expand caregiver problem solving and use of environmental solutions for problems with bathing and incontinence for elderly people with dementia	Randomised two group experimental pilot study Time period 3 months	Recruited spouse carers from a network of social service agencies ; Inclusion criteria: 1) reside with a spouse diagnosed with moderate Alzheimer’s Disease 2) provide assistance with 2 or more activities of daily living 3) serve as a primary source of care and 4) not receive any home care services. 37 spouse carers recruited and 17 randomly assigned (unspecified) to the treatment group. Carers: 15 Caucasian, 9 male, mean age 71. Co-resident. People with dementia: no details given Attrition: none	Intervention 5 visits by an occupational therapist (OT) using a framework from the competence –environmental literature. Visit 1 problem identification and review of current strategies, Visit 2 identification of environmental influences, education and development of plan. Visit 3 implementation of plan, Visit 4 expansion of plan, Visit 5 review and closure. Control An attention control group receiving home-making services.	Number of effective solutions offered by OT and used by spouse carer and evaluated as effective by OT for intervention group only.
Jirovec & Templin [27] 2001 USA Community	To determine if functional incontinence (FUI) could be reduced in memory impaired incontinent elders who had a individualised toileting programme (IST)	2 X 2 mixed design analysis by variance (group by time). Time period 6 months.	Recruited by advertisement for carers of ‘Memory impaired’ elders. Eligibility criteria not specified. 118 dyads recruited. 77 randomly assigned (by random number tables) to intervention (38 to bi-monthly follow up and 38 to 6 month follow up) and 41 to control. Memory impaired elders with FUI 82 females (69%) and 36 males (31%). Age: 79.89 (SD=7.93) yrs. c30% were African American. Interventions group mean SPMSQ [29] =6.64 (SD=2.2) and control group mean SPMSQ= 6.73 SD=2.44). FUI confirmed by assessment by nurse practitioner in patients home in consultation with an urologist. Carers: 79 females (67%), 39 males (33%). c30% were African American. 41% spouse, 39% adult child; remainder sibling, other relative or friend. Co-residence not specified. Attrition at 6 months 37%. 14 carers found the intervention ‘was too much for them’, 2 carers became ill or could not be reached. 19 elders moved to a nursing home. 9 people died.	Intervention 1. Individualised scheduled toileting (IST) agreed with carer. (unspecified if day and night) 2. Carers taught about age related bladder changes and 3) the importance of insuring adequate fluid intake 4. Home environments assessed for obstacles to urine control and advice given. 5. Pamphlet of teaching protocol , written at 6^th^ grade level left with carer 6. Monthly phone calls to review toileting schedule and difficulties, keep carers alert to intervention strategies, ensure carers offered elders 6–8 glasses of fluid a day and retain in study. 7. Bi-monthly visit (IST reviewed) or six month follow up visit. Control Control group paid $25 a visit. Monthly phone calls to maintain commitment to study and provide ‘friendly visits’	Percentage of time the patient was incontinent derived by dividing incontinence episodes by the total number of voiding episodes, both continent and incontinent. Baseline compared with follow up at 6 months All voiding episodes recorded by carer in continence diary kept for up to one week baseline and 6 months. Majority of carers only able to keep diary 3–4 days. During the same week, carers asked how often they were able to implement the IST protocol.
Enberg et al. [28] (2002) USA Community	To examine the short-term effectiveness of prompted voiding (PV) in cognitively impaired homebound older adults.	Exploratory study Prospective, controlled cross-over design where the usual care controls crossed over to the intervention following an 8 week observation period. Time period 10 weeks.	Participants recruited via Home Health Nursing Services Inclusion: > 60yrs, housebound, speak English, be incontinent at least 2x per week for at least 3 months and have a full time carer, MMSE [30] =<24. Exclusion: terminal illness; post void residual volume greater than 100 mL; their caregiver was unable or unwilling to participate or fewer than 2 incontinent episodes per week. 19 patient recruited and randomised by computerized minimization algorithm to the intervention (n=9) or control (n=10) Person with impairment. 68% female (n=13), mean age 83 yrs Mean MMSE[30]=17.24 (range 4–24) 95% needed help with bathing, 35% with eating and 79% needed assistance toileting. Carers (n=16, 8 for control and 8 for intervention) 69% female Mean age 65.2 (SD=12) yrs. All co-resident. Attrition: 3 of 9 intervention group before 8 weeks. 2 died and 1 carer became ill.	Intervention PV instruction to carers in 8 weekly sessions in patient’ homes by nurse practitioners (NPs). PV described as a behavioural therapy where carers approached patients every 2 hrs to ask if wet or dry, to check and to praise if dry and ask or encouraged to use the toilet. . PV every 2 hrs (daytime 12–16 hrs) but was individually modified. Carers also encouraged to stop caffeine drinks. If they had enuresis carers were advised to limit fluids in the evening. . Control The NPs visited every 1 to 2 weeks group to provide ‘socialization (attention control) of an average of 35 minutes without discussing incontinence. .	Percentage reduction in the average daily frequency of incontinent episodes Percentages of time subjects were wet by proportion of incontinence voids. Comparison of continence for the 2 weeks following the last control or treatment visit to the 2 week baseline period as recorded by the carers in bladder diaries. In addition for the carers a study designed questionnaire to assess perceptions of the intervention at the end.

All three studies were conducted in the United States of America (USA) and were published in 1993 [26], 2001[27] and 2002 [28]. Two studies described themselves as exploratory or pilot with small numbers of participants: 17 carers with 17 family members with dementia [26] and 19 patients and 16 carers [28]. The third study recruited 118 carer and patient dyads [27] but presented no explanation for the sample size.

The criteria and measures for dementia, cognitive impairment and incontinence varied. Gitlin and Corcoran [26] recruited spouse carers of people who were known to have a diagnosis of dementia and the incontinence problems were reported by the carer in an “unstructured interview” p14 [26]. Jirovec and Templin [27] recruited carers of “memory impaired elders” [27] p1 and assessed the mental status using the Short Portable Mental Status Questionnaire (SPQS) [29]. Incontinence was assessed by a study specific questionnaire answered by the carer and administered by a nurse practitioner, together with a physical examination, including bladder scan, of the person with dementia by the nurse practitioner. Endberg et al. [28] recruited patients and their carers who had been referred to the study by nurses from home health agencies, criteria unspecified at this point. Cognitive function was assessed at baseline by the Mini-Mental State Examination [30] and the Clock Drawing Test [31]. Incontinence was assessed by a study specific questionnaire administered by a nurse practitioner from the “subject” [28] p254 and the carer, together with a physical examination including bladder ultrasound.

All included studies were designed to compare outcomes between intervention and comparative groups. All interventions were educational or advisory and intended for carers to implement. Due to the nature of the interventions blinding of the participants or the professionals to the intervention in any of the studies was impossible. The interventions investigated were:

● An occupational therapist (OT) delivered intervention in five visits over three months to family carers. The intervention focused on problem solving for bathing and incontinence problems [26] for the carer to implement.

● A nurse practitioner (NP) delivered educational intervention with carers over 6 months, with a baseline visit followed by monthly phone calls and half also receiving bi-monthly visits. The NP with the carer planned an individualised toileting scheme (IST), which the carer implemented. The NP also provided continence education (such as adequate fluids), an educational leaflet and advice on environmental changes (e.g. leaving the bathroom light on at night) [27].

● A NP delivered prompted voiding (PV) instructional initiative to carers in eight weekly visits. In addition the NP gave advice to the carer on removing caffeine from the diet of the care recipient and restricting fluids in the evening if enuresis was a problem [28].

In two studies the control group received attention control only [26,27] and the third had a cross over design where after eight weeks the control group received the intervention [28].

Attrition rates varied. One study reported no attrition in three months [26]. The second study reported a 37% attrition rate (n=44 of 118 dyads) over 6 months. Reasons for attrition were that 19 people moved to a care home, 14 carers declined further participation or could not be contacted and ill health or death excluded a further 11 people [27]. In the third study three of the nine dyads in the intervention arm did not complete the study due to death or ill health [28].

The primary outcome measures were different: the number of OT offered solutions used by the family carer and judged effective by the OT (no measures for the control group) [26], the percentage of time the person with dementia was incontinent as reported in a carer continence diary completed for 3–4 days [27], the percentage reduction in the average daily frequency of incontinent episodes and the ‘percentage of time subjects were wet by proportion of incontinence voids’ [28 p 256] as reported in the carer completed daily bladder diary over eight weeks. Secondary outcomes were carer adherence to IST and PV agreed schedules [27,28].

The descriptive outcome data provided for the OT intervention [26] showed that 10 of 17 carers found incontinence problematic but only nine of the 17 OT initiated solutions for the carers to implement were acceptable. The least likely to be accepted was a toileting schedule. A secondary reported outcome was elimination of ineffective carer approaches to incontinence problems on the OT’s advice. Carers had ceased using ten of the fourteen observed, ineffective strategies e.g. shouting at the care recipient or not using protective undergarments at night, by the end of the study period. There was no discussion of the study’s limitations. This paper had a number of elements that contributed to a perceived high risk of bias, including the absence of any control group data (Table 3).

**Table 3:** Assessment of bias

**Domain**	**From the study**	**Review authors’ judgement**
**Giltin and Corcoran 1993**[26]		
Selection bias.	“randomly assigned to either attention control group who received home-making services or a treatment group” [26 p14] No data presented on the control group characteristics	Method of allocation not specified. No comment
Performance bias (blinding of participants and personnel)	Participants aware of receiving OT or home making service. OT aware they were providing the intervention.	Risk of bias
Detection bias (blinding of outcome assessment).	OT providing the intervention also provided the assessment of reported outcomes (care giver acceptance of solutions and elimination of ineffective care giver approaches).	Risk of bias
Attrition bias (incomplete outcome data)	No attrition from the study reported Data only provided for the intervention group on	Risk of bias
Reporting bias.	Reporting only on the intervention group.	Risk of bias
**Jirovec and Templin 2001**[27]		
Selection bias.	“Using a table of random numbers, volunteers were randomly assigned to either intervention or control group*”* [27 p2].	Low risk
Performance bias (blinding of participants and personnel)	Participants aware of receiving intervention or in control group. Personnel aware of those in the intervention or control group	Risk of bias
Detection bias (blinding of outcome assessment).	“The same person collected data from the intervention participants and the control group data collectors were not involved in the intervention”p5	Risk of bias
Attrition bias (incomplete outcome data).	Attrition rate, 37%, and reasons reported. “The loss of participants between the groups was not significantly different” [27 p5]. Three of four study measures reported. The implementation of IST by carers was not reported.	Low risk Risk of bias
Reporting bias.	The intervention arm was assigned into two groups, those that received bi-monthly visits and those that received a visit at 6 months. The data from these two arms were combined as the 6-month outcomes for percentage of time incontinent were “not significantly different” [27 p2] but not presented.	Risk of bias
Other points	The paper reports that this is a significant decrease in the experimental group using the non-parametric sign test (Z= −1.83, p<.05) [27 p 5]. As these figures appeared inconsistent we re-ran the sign test using the reported data which gave Z=−1.81, p=0.07 which is borderline but not significant. We re-ran the data on another version of the sign test, the exact binomial which gave a value of p=0.09 i.e. still not statistically significant between the groups.	
**Endberg et al. 2002**[28]		
Selection bias.	“Randomly assigned with use of a computerised minimisation algorithm” ( 28 p255) “Despite randomisation, the control group tended to have more severe incontinence than the treatment group” [28p259]	Low risk of bias
Performance bias (blinding of participants and personnel)	Participants aware of receiving intervention or in control group. Personnel aware of those in the intervention or control group	Risk of bias
Detection bias (blinding of outcome assessment).	“The 2 study NPs collected a comprehensive continence and medical history for the subject and caregiver” [28 p254] and provided the intervention and attention control.. “*at the end of the treatment the subjects were reassessed*” [28 p256]	Risk of bias
Attrition bias (incomplete outcome data).	*Three of 9 subjects randomly assigned to the treatment group dropped out or were excluded”*[28 p260] All outcomes and measures reported	Low risk of bias
Reporting bias.	All outcomes and measures reported	Low risk

The study investigating the IST instructional programme delivered by a nurse practitioner [27] was titled as a single intervention but involved multiple components including continence education and advice. This was initially a three arm study: two of intervention and one of control. The intervention arm was divided into two groups, those receiving bi-monthly visits and those receiving a visit at six months. Data from these two arms were combined because 6-month outcomes for percentage of time incontinent were “not significantly different” p2 [27]. No other data were presented for consideration of the three arms suggesting a risk of reporting bias (Table 3). Carers were reported as unable to complete the continence diaries for the entire requested week and the majority (unspecified) recorded voiding and incontinence for three to four days at baseline and at six months. Data was not presented as to the extent the carers were able to keep to the agreed IST. There was a reported mean reduction in incontinence frequency for those in the experimental arm as from 0.43 (SD 0.23) at baseline to a mean of 0.37 (SD 0.28) at six months compared to the control group with a mean incontinence frequency of 0.47(SD 0.31) at baseline to 0.49 (SD0.36) at 6 months. The authors reported a decrease (unspecified amount) in incontinence at 6 months compared to baseline in 28 of 44 participants in the experimental group and in 15 of the 30 control group, specified as a small amount. Using the non-parametric sign test they reported a significant decrease in the experimental group (Z= −1.83, p<.05), p5 [25]. As this appeared inconsistent with the data, the sign test was re-run by RG using the reported data p5 [25]. The results of this re-analysis by RG are given in Table 3. The results were found to be borderline but not statistically significant.

The third study, described as exploratory, investigating the effectiveness of the nurse practitioner (NP) delivered prompted voiding (PV) instruction to 16 carers for 19 people with dementia [28]. Nine patients and their carers were randomly assigned to the intervention and six completed the entire eight weeks. Ten patients were assigned to the attention control group and crossed over to receive the intervention. There was a low risk of bias in those elements the researchers could address (Table 3). Carers were adherent to the intervention for an average of 89% of the time (SD =10.4, range 71 to 100%). People with dementia responded to prompts to go to the toilet on an average of 76% of the time (SD =34%, range 8% to 100%). Using the intention to treat approach, there were no statistical differences reported between the treatment group and the control group for any of the UI outcomes measured. Analysis of data for all 15 people with dementia reported the mean number of daytime incontinent episodes decreased from 2.2 (SD=1.4) per day at baseline to 1.8 (SD 1.6) post intervention (22% reduction *t*=1.8, *P*=0.4,) [28 p260]. However, while ten people were found to have a decrease in incontinent episodes, five were reported to have an increase. This study was the only one of the three to systematically explore the impact and satisfaction of the carers with the intervention though validated scales [32] and a study designed questionnaire. Seven of fifteen carers reported the intervention had decreased their caring workload, three that it had remained the same and five that it had increased their workload. The limitations of the study were discussed with regard to the small sample size with the power to only detect very large differences between the treatment and control groups. The authors concluded that there were clinically significant reductions in UI for many of the people with dementia although the findings were not generalisable.

## Discussion

Only three intervention studies were identified in the review, two were exploratory or pilot studies and all three had some methodological weakness resulting in bias. In all the findings are described as tentative and additional research is required. None of the studies investigated outcomes related to costs or the quality of life of either the carer or the person with dementia although all did throw light on the acceptability and feasibility of interventions implemented by carers. All three studies focused on urinary incontinence, dated from over a decade ago and had educational and advice interventions to be implemented by carers. One study suggested that a tailored intervention could reduce ineffective strategies in managing continence but recommended this required further investigation [26]. The study investigating a multi-component, educational IST recruited the largest sample and drew conclusions of a significant effect. However; a re-analysis of the data by the authors of this paper did not replicate this [27]. This raises questions as to the rigour of the analysis and the conclusions. The third study concluded that PV education (including eight weekly visits by NPs) implemented by family carers could make significant clinical reductions in UI for many people with dementia [28] but the limitations of the study suggest further testing is required.

All three studies illuminate the issue of acceptability and feasibility of interventions to carers. While the viewpoint of the person with dementia was not investigated, one study noted that most failures to adhere to the toileting schedule were a result of resistance on the part of the person with dementia [27].

To our knowledge this is the first systematic review addressing the question of the effectiveness of conservative interventions for incontinence in this population, resident at home. Other published reviews have either not specified the setting (although much of the evidence presented in them relates to care homes only) [33,34], or present clinical expert opinion rather than a systematic review of research evidence [35]. Reviews have also been undertaken of behavioural interventions for urinary incontinence but include studies for both cognitively intact and also impaired individuals, and those resident at home as well as in care homes [36,37], making it impossible to identify the impact on the population of interest here.

All three included studies suggested that further research was required but no further studies were identified in the intervening decade. The reasons for the dearth of published research deserve consideration. In 2010 the International Continence Society (ICS) Committee for the Frail Elderly [19], of whom some people with dementia are one sub group, noted “the continuing paucity of clinical trials” (p165) in this population. The ICS Committee argued that the management of urinary incontinence in the frail elderly must be multi-component, address co-morbidities, and take cognisance of other impairments, and of preferences. A recent United Kingdom (UK) retrospective cohort study reported on the effect of comprehensive geriatric assessment (CGA) combined with such a multi-component management approach to incontinence in 112 frail, community dwelling patients, of whom 30% had dementia [38]. While the data for those with dementia are not presented separately, the authors report that dementia was not associated with poorer treatment outcomes [38]. The applicability of such approaches by specialist and generalist health care professionals caring for people with dementia living at home requires further investigation.

The challenge for clinicians and researchers working in community and primary care settings is to design and undertake investigations that test multi-component interventions for this population. The Medical Research Council guidance for developing and evaluating complex interventions offers a helpful stepwise framework [39]. The studies reviewed here point to issues which need specific consideration: difficulties in recruitment, issues in acceptability for family caregivers and last but not least the perspective of feasibility and acceptability for the person with dementia, which is absent in all three studies [26-28]. Feelings of embarrassment and stigma, associated with both incontinence and dementia [16,40] are likely to negatively impact on recruitment to such studies.

Well constructed research takes time to conduct and report. The immediate issue for doctors, nurses and other health professionals working with people with dementia and their family carers is how to draw on best evidence in developing and advising on management plans. The ICS committee for frail elderly people offers valuable principles and summarises current evidence for that group [19] but it has limitations for this sub-group in that it: a) does not address the range of toileting and incontinence problems experienced by people with dementia at different points in the course of the disease [10,14] and b) it is sometimes difficult to separate the recommendations relevant to those living at home from those living in care homes. This is true of other current guidelines such as the American Academy for Neurology (AAN) guidelines for the management of dementia which states that “scheduled toileting and prompted voiding reduce urinary incontinence” [41] p1 in people with dementia and English National Institute for Health and Clinical Excellence (NICE) recommends that in the behavioural management for urinary incontinence in people with neurological conditions “prompted voiding and habit re-training are particularly suitable for people with cognitive impairment” p35 [42]. Both have examined and cited evidence from studies conducted in care homes. Neither makes it clear this may not necessarily apply to those who are resident at home. We suggest there is a gap that urgently needs to be addressed by an expert group, including carers, to develop guidelines based on consensus methods.

The review has limitations. Exclusion criteria such as reporting in English only may have resulted in the omission of studies. In addition, the review may not have identified studies where the intervention was more broadly focused and reported results on incontinence amongst other outcomes. However, the search strategy, using carefully selected search terms, was designed to be as wide as possible to mitigate such problems.

## Conclusions

Incontinence problems in people with dementia have a significant impact for the individual, their families and the broader health system. This review identified only three reported studies investigating conservative interventions for urinary incontinence and none provided evidence to support or rule out the effectiveness of these interventions. Each provided insights into aspects such as acceptability to carers that can help shape multi-component interventions for future testing. The lack of research and focused attention on these problems in people with dementia or cognitive impairment, living at home, is evident through other reviews and clinical guidance which fail to differentiate between those living at home and those living in care homes. In the face of growing numbers of people with dementia, there remains an urgent need for both research and clinical guidance for health professionals tailored to the setting where the majority of people with dementia live.

## Competing interests

The authors declare that they have no competing interests.

## Authors’ contribution

VMD conceived the study and with SI and GR obtained funding. VMD and NG with advice from GR, MF and SI designed the study. NG, LC and VMD conducted searches, study selection and data extraction. NG, LC, RG, VMD conducted analyses advised by MF and GR. VMD prepared the first draft. All revised drafts for important intellectual content and agreed the final content of the paper.

## Pre-publication history

The pre-publication history for this paper can be accessed here:

http://www.biomedcentral.com/1471-2318/12/77/prepub

## Supplementary Material

Additional file 1 Search Strategy on database Ovid Medline. Click here for file
