# Supplementary material for: Conservative interventions for incontinence in people with dementia or cognitive impairment, living at home: a systematic review
Source: BMC Geriatr. 2012 Dec 28;12:77. doi: 10.1186/1471-2318-12-77 (PMC3562513; doi:10.1186/1471-2318-12-77)
Supplement: Additional file 1 — Search Strategy on database Ovid Medline. [file 1471-2318-12-77-S1.pdf]

Additional File 1
Search Strategy on database Ovid Medline (R)

Database: Ovid MEDLINE(R) <1950 to April Week 12 2012>
Search Strategy:

-----

1 exp Fecal Incontinence/ or exp Urinary Incontinence/ (23883)
2 exp Delirium, Dementia, Amnestic, Cognitive Disorders/ or exp Dementia/ (119192)
3 dementia.mp. (57996)
4 2 or 3 (127165)
5 1 and 4 (404)
6 exp Aged/ (1670832)
7 5 and 6 (307)
8 exp caregivers/ (11312)
9 exp spouses/ (3768)
10 exp siblings/ (2639)
11 exp parents/ (47229)
12 exp family/ (170550)
13 exp home care services/ (32359)
14 exp community health services/ (367707)
15 exp home nursing/ (7636)
16 exp social support/ (30570)
17 exp voluntary workers/ (6642)
18 exp toilet facilities/ (833)
19 exp home health agencies/ (898)
20 exp homemaker services/ (420)
21 exp homebound persons/ (314)
22 or/8-21 (540067)
23 7 and 22 (37) - see
24 exp occupational therapy/ (7676)
25 exp activities of daily living/ (33547)
26 (activities of daily living or adl).mp. (37579)
27 exp self care/ (26176)
28 exp diapers, adult/ (40)
29 exp incontinence pads/ (305)
30 exp toilet training/ (627)
31 exp attitude to health/ (181810)
32 exp adaptation, psychological/ (70252)
33 exp needs assessment/ (14060)
34 exp Behavior Therapy/ (35553)
35 exp ambulatory care/ (37683)
36 or/24-35 (376283)
37 7 and 36 (106)
38 37 not 23 (82) - see
39 7 and manag$.mp. [mp=title, original title, abstract, name of substance word, subject heading word] (42)
40 39 not (37 or 23) (15) - see
